# Costs saved and avoided from pharmacist interventions to address drug-related problems identified from outpatient clinics in Jordan

**DOI:** 10.1371/journal.pone.0302287

**Published:** 2024-06-06

**Authors:** Ghaith M. Al-Taani, Suhaib M. Muflih, Sayer I. Al-Azzam, Karem H. Alzoubi

**Affiliations:** 1 Faculty of Pharmacy, Department of Clinical Pharmacy and Pharmacy Practice, Yarmouk University, Irbid, Jordan; 2 Faculty of Pharmacy, Department of Clinical Pharmacy, Jordan University of Science and Technology, Irbid, Jordan; 3 Department of Pharmacy Practice and Pharmacotherapeutics, College of Pharmacy, University of Sharjah, Sharjah, UAE; 4 Faculty of Pharmacy, Jordan University of Science and Technology, Irbid, Jordan; King Saud University, SAUDI ARABIA

## Abstract

**Background:**

The pharmacist plays an essential role in identifying and managing drug-related problems. The aim of this research was to assess the costs avoided by clinical pharmacist interventions to resolve drug-related problems.

**Research design and methods:**

Clinical pharmacists identified drug-related problems and interventions to address them in consecutive outpatients visiting internal medicine clinics at major teaching and public hospitals in Jordan from September 2012 to December 2013. The costs avoided by each intervention to address drug-related problems were collected from the literature. The collected data were used to calculate the overall cost saved and avoided by the interventions implemented to address the identified drug-related problems, adopting a Jordanian healthcare system perspective.

**Results:**

A total of 2747 patients were enrolled in the study. Diagnostic interventions, such as the need for additional diagnostic testing, were employed in 95.07% of the 13935 intervention to address the drug-related problem “Miscellaneous” which was the most frequent drug-related problems. Other common drug-related problems categories included inappropriate knowledge (n = 6972), inappropriate adherence (4447), efficacy-related drug-related problem (3395) and unnecessary drug therapy (1082). The total cost avoided over the research period was JOD 1418720 per month and total cost saved over the study period was JOD 17250.204. Drug-related problems were associated the number of prescription medications (odds ratio = 1.105; 95% confidence interval = 1.069–1.142), prescribed gastrointestinal drugs (3.485; 2.86–4.247), prescribed antimicrobials (3.326; 1.084–10.205), and prescribed musculoskeletal drugs (1.385; 1.011–1.852).

**Conclusions:**

The study revealed that pharmacists have provided cognitive input to rationalize and optimize the medication use and prevent errors, that led to the reported projected avoided and saved expenditures via various interventions to address drug-related problems. This highlights the added economic impact to the clinical impact of drug-related problems on patients and the healthcare system. The high prevalence and cost of drug-related problems offer strong rationale for pharmacists to provide more vigilant intervention to improve patient outcomes while maintaining cost effectiveness.

## Introduction

The improvement of a person’s health and wellbeing is one of pharmacotherapy’s primary objectives, and drugs are the most common healthcare intervention [1]. In normal care, a patient’s outcome may be compromised in relation to medication, and this is known as a drug-related problem (DRPs). DRP is defined by Pharmaceutical Care Network Europe (PCNE) as, “an event or circumstance involving drug therapy that actually or potentially interferes with desired health outcomes” [2]. This definition is almost the same as the definition of the seminal paper, “Opportunities and Responsibilities in Pharmaceutical Care” [3]. Pharmaceutical care is delivered by pharmacists to rationalize the medications used by patient, a cornerstone activity in pharmaceutical care is services to address DRPs [4]. The centeredness associated with DRPs services dictate that services should be delivered with rapport and to engage patients in the decision making, goal setting, and monitoring for therapies [5]. Pharmaceutical care need has been conceptualized at the end of the last century, which has been reflected on the wide adoption of expanded role of the pharmacists [3,6].

On average, hypertensive and diabetic patients could suffer from 5–6 different DRPs in outpatient clinics in Jordan [7,8]. Many DRPs can be encountered, including: inappropriate drugs, unnecessary drugs affecting older patients, no indication for the drug, the drug dose being too high, and untreated indication in general practice [9,10].

With limited resources, pharmacoeconomic analysis is needed to estimate the financial implications of the healthcare interventions, whilst preserving the best outcomes. In addition, identification, valuation, and estimating the monetary effect associated with DRPs is important to shed light on the outcomes associated with DRPs, facilitate DRPs reduction services and emphasize the role of pharmacists [11]. More outcomes that can be associated with unaddressed DRPs are drug-related mortality and associated costs [12]. In other cases, potential DRPs may be present, whilst not currently causing problematic outcomes, However, these DRPs could affect a patient in the future if left unaddressed.

A number of studies that has been carried in Jordan assessing the cost of DRPs and these studies were focused on hospitalized patients and other focused groups of patients, i.e. asthma [13,14]. In Jordan, a number of challenges have been identified within the pharmacy sector, including the high incidence of DRPs in a community setting, low provision of services to address DRPs by community pharmacists, low consultation time from physicians and low dispensing time of pharmacies [15–17]. Based on these observations, it is important to have convincing evidence of the value that pharmacists provide to address DRPs. Thus, the aim of the present study was to analyze the amount of costs avoided by clinical pharmacists’ interventions to address drug-related problems.

## Methods

The present study was a cross-sectional, prospective study that reviewed patients’ medicines for the presence of DRPs. Interventions proposed and cost avoided were the focus of the present study.

### Setting

The present study was carried out in a number of hospitals in Jordan over the period 1 September 2012–31 December 2013. These hospitals included King Abdullah University Hospital (teaching hospital in the north of Jordan affiliated by Jordan University of Science and Technology); Al-Basheer Hospital (public hospital in the capital of Jordan); Prince Hamza Hospital (public hospital in the capital of Jordan), Princess Basma Hospital (public hospital in the north of Jordan) and Al-Karak Hospital (public hospital in the south of Jordan). Ethical approval to carry out the present study was granted from the research ethics committees that oversee these hospitals, namely the ministry of health research ethics committee and King Abdullah University Hospital research ethics committee (reference: 22/5/2012).

### Patient population

Consecutive patients attending cardiology, respiratory, and endocrine outpatient clinics in a number of hospitals in Jordan were screened for eligibility to take part of the study. Eligible patients are those patients 18 years of age and more, received at least two long-term medications, and have one medical condition or more. Exclusion criteria included patients who cannot give informed consent, e.g., mental illness, and those who don’t speak Arabic. According to the sampling framework of who are attending outpatient clinic visit, those patients who met the selection criteria were approached and information regarding the study and what does it involve was provided while waiting for their outpatient appointment and those who agree to take part were asked to provide written informed consent to take part in the study and to allow data from their medical records to be used in research.

### Study procedure

Recruited patients’ medication regimens currently used by the participants were reviewed for the presence of DRPs by pharmacists with Doctor of Pharmacy degrees and who were trained on the study procedure. Those pharmacists work full time in the hospital and develop, in collaboration with multidisciplinary team, pharmaceutical care plan for individual patient as routine day-to day-activities. Demographics, clinical data, identified DRPs and their interventions, and linked drug and disease were documented [15]. The sample size calculation revealed that based on a margin of error of 5% with 95% confidence level a minimum sample size required is 383 participants.

### Definition of DRPs

The DRPs identified in the present study were categorized in the following categories: unnecessary drug therapy (e.g., drug use without and indication), untreated condition, efficacy (e.g., efficacy interaction issue), safety (e.g., allergic reaction or an undesirable effect), inappropriate knowledge (e.g., the patient is not instructed), inappropriate adherence and miscellaneous (e.g., the need for additional of more frequent monitoring). These categories are based on the AbuRuz et al., classification system, and included a comprehensive list of DRPs and was utilized in similar other published work in secondary care setting [18–20]. These DRPs were recorded if present one by one, and it is possible that one prescription has multiple DRPs. As these DRPs were recorded, the clinical pharmacists provided interventions to address these DRPS and were quantified, including the total number of each intervention was recorded and the percentages of different categories of interventions were reported. The categories of interventions included no intervention, patient-related (counseling and education initiatives), drug-related (change drug, stop the drug, start a new drug, change the dosage, change instruction and drug level), monitor (following up patients to assess achievement of therapeutic outcomes or development of adverse effects), refer to other healthcare professionals for the best interest of the patient, diagnostic (i.e., the need for additional diagnostic testing), knowledge (address a problem in medicines knowledge such as related to working knowledge of the name of medication, indication or dose regimen) adherence (emphasize adherence which was formally assessed) and consultation (require further review)

### Data analysis

Standard statistical methodologies were used in the estimation of the prevalence, costs avoided, and risk factors for the presence of selected DRPs, using Statistical Package for Social Sciences (SPSS) version 26. Descriptive statistics were used to summarize the data. The Chi-square test was used to determine the association of categorical variables and patients with DRPs versus those without DRPs. A number of analytical approaches were carried out in the present study:

#### a. Estimation of the cost avoided from DRPs

Cost avoidance refers to the cost saved via prevention of utilization of healthcare resources due to potential drug-related problems by pharmacist recommendation, this cost is hypothetical that represent cost incurred with the absence of pharmacist recommendation. Costs avoided from interventions to address DRPs were calculated via counting the numbers of different categories of DRPs interventions and applying the estimated cost avoided per month per intervention for different categories of DRPs. The average cost avoided per intervention were obtained from the research literature [21,22]. In the latter study, the types of DRPs and frequencies was collected via pharmacist-led medication review in the outpatient clinics setting in Jordan within close time period to our study and the post hoc probability of harm and cost avoided of prevented negative consequences of DRPs was obtained as a result a specialized experienced assessment panel, utilizing the mean costs for emergency department visits and internal medicine admission [21,22]. They undertake the approach of Nesbit et al [23]. Total costs for each category of DRPs were the result of multiplication of the frequency (n) of the DRPs category by average cost avoided per intervention per month (JOD). A total cost avoided by interventions to address DRPs from the present study was the summation of the costs of each category of DRPs, which is different from that from the USA [24].

#### b. Estimation of cost saved by the interventions to address DRPs

Cost saving refers to the financial benefits drawn by pharmacist interventions to optimize treatment that equals the actual reduction drug expenditure via recommendation to discontinue a medication. An estimation of costs saved from pharmacist intervention was carried out, considering the drug cost using data from national resources, which was the Joint Procurement Department (JPD) at the time of data collection (available at https://gpd.gov.jo/). The estimation of cost saved was based on the cost of implicated drug with different categories of DRPs, in which an intervention that involve the drug was carried out (e.g., intervention to discontinue a drug). Different categories costs were summed up that in three main categories of DRPs, namely unnecessary drug therapy, efficacy-related DRPs and safety-related DRPs. A total cost saved by interventions to address DRPs was the result of the summation of the aforementioned three categories of cost of DRPs.

#### c. One-way sensitivity analysis

A sensitivity analysis was carried out to test the variability on different uncertain variables on the total costs avoided (base case). It was represented graphically using a tornado diagram. Each uncertain variable was associated with a bar in which the width of the bar represents the impact that factor has on the total cost. For each uncertain variable, minimum and maximum values were estimated. In the one-way sensitivity analysis, one variable is variated (into minimum and maximum values) and all other variables remained unchanged.

#### d. Independent predictors for DRPs using logistic regression analysis

A number of potential variables that have the potential to serve as predictors for DRPs were investigated. These variables included: age, sex, education, occupation, marital status, health insurance, number of medications, adherence to drugs, adherence to self-care and nonpharmacological recommendation, medication knowledge, and drug class according to British National Formulary [25]. An initial univariable analysis was carried out to determine the candidate variables, using the chi-square test and independent samples t-test depending on the type of variable. The candidate variable to be included in the logistic regression model was defined at p≤0.250. Candidate variables selected were entered in a backward logistic regression model utilizing the presence of DRP as the outcome variable. Only statistically significant, independent predictors associated with outcome variables were retained in the final model. Statistical significance was set as p≤0.050.

## Results

The number of patients included in the study was 2747 patients. The most common types of interventions were linked to the following DRPs categories including miscellaneous (e,g., a need for additional or more frequent monitoring) (13935 interventions), inappropriate knowledge (6972interventions), and efficacy (3395intervention). A low number of interventions were proposed to address untreated condition (3 interventions). Table 1 Summarizes the relationship of a number of demographic variables with DRPs, that the present study focused on. Those aged less than 40 years old were more likely to have no DRPs, whereas age more than 40 years old was associated with DRPs (p<0.001). More females (approximately 60%) were recruited than males (40%). Approximately 80% of the study sample were married. Secondary education and further was associated with no DRPs, on the other hand, primary education and less were associated with DRPs. Most of the sample size were unemployed, despite this, the overwhelming majority of them (about 96%) were covered with health insurance.

**Table 1:** Demographic characteristics of the study sample by DRPs present.

variable	Categories	No DRPs [n (%)]	DRPs present [n (%)]	p value
Age (years)	25 or less	41 (3.1)	16 (1.1)	<0.001
	26–40	121 (9.1)	60 (4.3)	
	41–65	862 (64.8)	932 (66.1)	
	More than 65	306 (23.0)	403 (28.6)	
Sex	Male	540 (40.7)	588 (41.8)	0.560
	Female	788 (59.3)	820 (58.2)	
Marital status	Single	249 (18.7)	254 (18.0)	0.620
	Married	1082 (81.3)	1159 (82.0)	
Educational level	Illiterate	200 (15.0)	247 (17.5)	0.001
	Primary	355 (26.7)	450 (31.8)	
	Secondary	398 (29.9)	399 (28.2)	
	Community College	172 (12.9)	160 (11.3)	
	BSc	183 (13.8)	135 (9.5)	
	Post-graduate degree	21 (1.6)	23 (1.6)	
Occupation	Jobless	980 (73.6)	1104 (78.1)	0.022
	Medical	18 (1.4)	17 (1.2)	
	Non-medical	333 (25.0)	292 (20.7)	
Health insurance	Insured	1280 (96.4)	1372 (97.0)	0.344
	Non insured	48 (3.6)	42 (3.0)	

It seems that the clinical pharmacists intervened extensively. The interventions were linked to the category of DRPs. For example, diagnostic-related interventions were utilized in 95.07% of the 13935-linked DRP, miscellaneous. Common DRPs identified included miscellaneous (n = 13935), inappropriate knowledge (n = 6972) and Inappropriate adherence (n = 4447). Common interventions utilized included patient related which was commonly (87.43%) utilized for the DRP unnecessary drug therapy, knowledge which was commonly (99.13%) linked to the DRP inappropriate knowledge and adherence which was commonly (87.81%) linked to the DRP inappropriate adherence. Full details are shown in Table 2.

**Table 2:** Different types of interventions proposed for different categories for DRPs.

DRPs category	n	No intervention (%)	Patient related (%)	Drug related (%)	Monitor (%)	Refer (%)	Diagnostic (%)	Knowledge (%)	Adherence (%)	Consultation (%)
Unnecessary drug therapy	1082	2.13	87.43	0.09	5.18	4.16	0.00	0.00	0.00	0.18
Untreated condition	3	100.00	0.00	0.00	0.00	0.00	0.00	0.00	0.00	0.00
Efficacy	3395	42.50	3.98	41.65	1.27	7.57	0.29	2.12	0.71	0.00
Safety	346	8.96	28.03	13.87	13.58	22.54	11.56	0.87	0.29	0.29
Inappropriate knowledge	6972	0.01	0.00	0.00	0.00	0.00	0.62	99.13	0.19	0.06
Inappropriate adherence	4447	0.00	0.00	0.00	0.00	0.00	0.16	12.01	87.81	0.02
Miscellaneous	13935	0.00	0.04	0.00	0.00	0.01	95.07	0.38	0.02	4.48

Regarding the cost avoided from identified DRPs. The highest costs avoided and would be incurred if DRPs are not identified and addressed were related to the DRP category inappropriate knowledge which was JOD 431,385.5. Other categories of DRPs associated with high costs included inappropriate adherence (JOD 321,011.1) and miscellaneous DRPs JOD 292,189.1). The total cost avoided through the study period was JOD 292,189.1. In relation to the “cost saved” from the pharmacist intervention for the whole study period, efficacy-related DRPs were associated with highest cost saved (JOD 12,192.987), followed by unnecessary drug therapy (JOD 4,119.226), then safety-related DRPs (JOD 937.991). The total cost for saved for the whole study period by the clinical pharmacist intervention that included the sum of the three aforementioned costs saved was estimated to be JOD 17,250.204Full details regarding the costs avoided from identified DRPs are illustrated in Table 3.

**Table 3:** Costs avoided and saved from identified DRPs.

DRP category	“Cost Avoidance” per month according to the costing study (JOD)	“Cost avoidance” per month per DRP according to costing study (JOD)	Number of DRPs in the present study	Total cost avoided per month in the present study (JOD)	“Cost saved” for the whole study period (JOD)
Unnecessary drug therapy	794.04	158.808	1082	171830.3	4119.226
Untreated condition	288.74	57.748	3	173.244	-
Efficacy	866.23	50.955	3395	172992.2	12192.987
Safety	505.29	84.215	346	29138.39	937.991
Inappropriate knowledge	866.23	61.874	6972	431385.5	-
Inappropriate adherence	794.04	72.186	4447	321011.1	-
Miscellaneous	440.34	20.968	13935	292189.1	-
Total				1,418,719.834	17250.204

**Fig 1:**
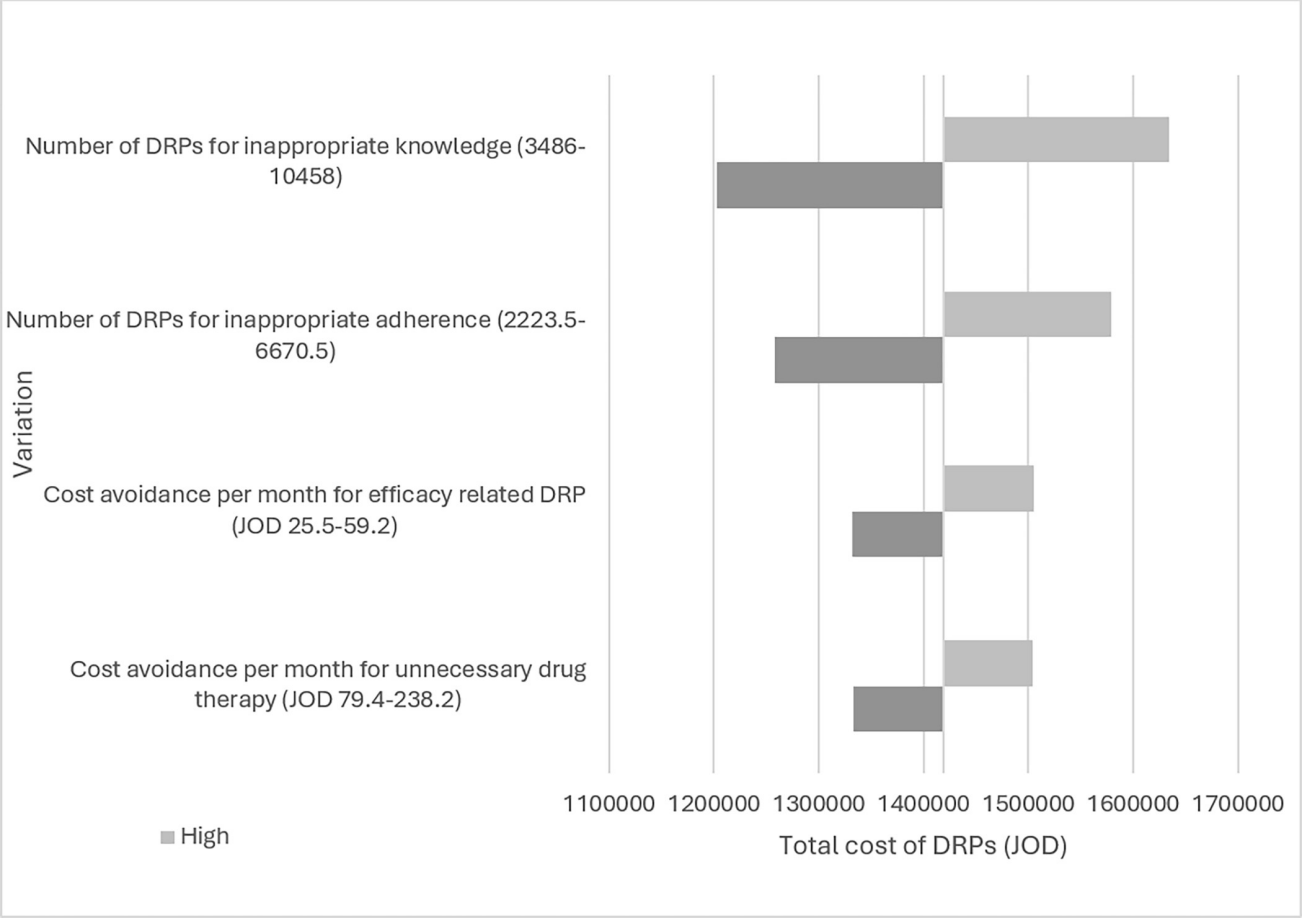
One-way sensitivity analysis for the estimated costs avoided from pharmacist intervention to address DRPs represented via tornado diagram.

The results of the one-way sensitivity analysis are represented in Fig 1. The high level of number of DRPs for inappropriate knowledge was responsible for most of the highest avoidable costs associated with DRPs (JOD 1,634,412.62). Table 4 summarizes the independent predictors associated with having a DRP (including the odds ratios). The final predictors included increased age (>25 years old), number of medications prescribed (odds ratio = 1.105), prescribed gastrointestinal drugs (BNF category; odds ratio = 3.485), prescribed drugs for infection (BNF category; odds ratio = 3.326), and prescribed musculoskeletal drugs (BNF category; odds ratio = 1.368).

**Table 4:** Independent predictors associated with DRPs.

Predictor	B (S.E.)	p values	Odds ratio (95% C.I.)
Age (25 years or less)		0.019	
Age (26–40 years)	0.084 (0.349)	0.81	1.087 (0.549–2.153)
Age (41–65 years)	0.519 (0.314)	0.098	1.681 (0.909–3.108)
Age (More than 65 years)	0.595 (0.322)	0.064	1.814 (0.966–3.407)
Number of medications	0.1 (0.017)	<0.001	1.105 (1.069–1.142)
Gastrointestinal drugs	1.248 (0.101)	<0.001	3.485 (2.86–4.247)
Drugs for infections	1.202 (0.572)	0.036	3.326 (1.084–10.205)
Musculoskeletal drugs	0.313 (0.155)	0.043	1.368 (1.011–1.852)
Constant	-1.372 (0.311)	<0.001	0.254

B: Unstandardized regression weight.

S.E.: Standard error.

## Discussion

DRPs in Jordan are more likely to occur in an outpatient setting as opposed to a hospital setting due to the high rates of DRPs in the outpatient setting [15] and the limited provision of intervention to address DRPs by community pharmacists [16]. This emphasizes the necessity of research output informing the value and importance of pharmacist input to enhance the management of outpatient medications. Given the limited amount of healthcare resources available, justification based on pharmacoeconomic evidence is crucial. The present study attempted to identify and categorize the interventions to address DRPs and value the costs avoided by the interventions to address DRPs in a sample of patients attending outpatient clinics. Pharmacists intervened actively. The most common type of DRPs identified was miscellaneous category (n = 13935) and patient-related intervention was commonly (87.43%) utilized for the DRP unnecessary drug therapy. The estimated avoided costs for the attendees of study outpatient clinics per month would be JOD 1,418,720. Increased age, increased number of medications and prescribed gastrointestinal, infectious or musculoskeletal drug are predictors of DRPs.

The results highlight the benefits of pharmacist services to address DRPs in outpatient settings. Such benefits strengthen the societal need for services to address DRPs, a serious avoidable economic problem.

It seems that certain demographics were associated with DRPs, e.g., age, educational level, and occupation. Increased age, i.e., elderly, was associated with DRPs, as well as having secondary education and higher was associated with low DRPs. The results highlight those elders are more likely to have polypharmacy and age-related changes in pharmacokinetics and pharmacodynamics that increase the odds for DRPs. Geriatrics utilize many of the healthcare resources and the majority of elderly patients suffer from DRPs.[2] It is recommended to pay attention to this group of patients when designing DRPs services, particularly those with multimorbidity and polypharmacy. Whereas secondary and tertiary education was found less associated with DRPs and this is related to that this group expected to have higher health literacy compared with participants with primary education or illiterate. Indeed, it has been reported that education below high school was associated with hospitalization implicated by DRPs [26].

It seems that many interventions were proposed, that indicate that high number of DRPs present within the outpatient setting. The control of the use of medication particularly in administration moves to patients in an outpatient setting. Despite this, the high level of DRPs is not related to patient management of medications but is most probably a care issue from the healthcare professionals. Most problems identified are related to need for monitoring (part of miscellaneous DRPs) and inappropriate adherence and knowledge. The increased number of medications and complexity of healthcare make medication management by practitioners difficult. Thus, increased enhanced clinical pharmacy services is important in outpatient clinics, which to date is not provided, and as evident in the present study can help reduce the number of DRPs, particularly serious DRPs, with reduced cost to the healthcare system. Within the outpatient setting many interventions were provided by pharmacists [27–29], which provides evidence based on the importance of increased pharmacist input to address DRPs within this setting, particularly in developing countries. Medication therapy management services provided to patients in community pharmacies in the United States are considered a model that provides pharmaceutical care in routine service. This model has been scrutinized and optimized through cumulative experience with medication therapy management services [30,31].

Common DRPs identified by the clinical pharmacist review included need for monitoring (part of miscellaneous DRPs) and inappropriate adherence and knowledge. Such DRPs are considered a priority in outpatient settings and pharmacists and other healthcare professionals should remain vigilant to these DRPs. Published research has found many DRPs occur in different settings [32–34]. These include adverse drug events and medication errors,[35] as well as they, have a prevalence of 18% in outpatient settings [36]. Interventions provided by clinical pharmacists further document the need for further service to address DRPs in the outpatient setting routinely. This can be achieved via a collaborative approach with other healthcare professionals to address issues such as changing drugs, stopping drugs, starting new drugs… etc. Also, of benefit in this regard would be adopting the patient-centered approach in which the pharmacist can work directly with the patient to counsel, educate and increase the knowledge of the patient regarding his disease and medications and trying to consider the patient preferences within the therapeutic plan [37,38].

The DRPs and associated costs to the healthcare system are important in the resource-scarce healthcare system. Such data supported the case of the need for clinical pharmacy services in the outpatient setting. The cost per one month was JOD 1,418,720 which is not insignificant and highlights that many of the costs of care for patients can be related to untreated and unidentified DRPs. The development of clinical pharmacy services in the outpatient setting is highly needed. An estimation of the cost of drug-related morbidity and mortality in 2000 using the decision-making model was $177.4 billion, mostly due to hospital admission (70%) [12]. It is not known how much is spent on medications in the outpatient setting and in nursing facilities it was estimated that when there is a spent of $1 on medications an additional $1.33 are spent on DRPs from different healthcare resources [39]. Excess costs associated with DRPs are not identified easily through a literature review, it seems that an adverse drug event associated with an avoidable cost of $4685 as it is estimated to lead to increasing in-hospital stay [40]. These costs provide an indication of the effects of the DRPs, however, these figures are not ready for use in the Jordanian health system and are not considered recent statistics.

Independent predictors associated with DRPs on the multivariate analysis provided an insight into the group of patients that DRP-reduction services should focus on. Such targeting is an important initial step in the development of interventions to address DRPs. Increased age increased the odds of DRPs due to the fact that this group of patients is more likely to have polypharmacy. In addition, polypharmacy on its own is associated with DRPs. Polypharmacy was an important risk factor for DRPs in the multidisciplinary triangulation process [41]. A number of drug groups including gastrointestinal, infection, and musculoskeletal drugs were associated with DRPs on multivariate analysis. Possible reasons for the appearance of these medications as predictors can be related to the comorbidities they represent, the risk associated with these medications (considered high alert medications), and the self-use of these medications by the patients without consultation with a physician. Indeed, gastrointestinal medications were associated with important DRPs through a predictive model [42]. Pharmacists and other healthcare professionals should keep an eye on these predictors in order to develop a successful medication management service to address DRPs.

Despite that currently no centralized patient records present in Jordan, the technological integration in healthcare has received momentum, particularly with the implementation of centralized or interconnected healthcare administration system (Hakeem) which was launched in 2009, with enthusiastic hopes to be an e-health system of national health records [43]. Also, the electronic library of medicine (ELM) was another governmental initiative in Jordan that provide free access to many evidence-based resources and references, including medical books, scientific journals and other resources, that aim to incorporate evidence-based direct patient care, safeguarding best possible patient outcomes [44]. In the regulatory aspect, since 2012 it has been required for drug pricing to conduct an economic analysis and it is expected that pharmacoeconomic evidence to inform formulary addition [45]. The health care system in Jordan has witnessed unavoidable challenges, including the arrival of Syrian refugees, albeit some organization of medical care was provided by United Nations High Commissioner for Refugees (UNHCR), the capacity of the present healthcare system and the society was unable to meet their health needs and, as such, they were considered a burden on the healthcare sector [46].

The current study gave research proof of the pharmcoeconomic impact of clinical pharmacy services, which can stimulate the discussion on the significance of putting DRPs-reduction services in implementation. A reasonable sample size, a prospective, multicenter study, and the fact that few similar studies carried out in Jordan highlight the proof the present study is providing. The present study is limited, despite the data collection was done by trained pharmacists and accompanied by an implementation procedure, there is a chance of inter-rater variation, however it can indicate a component of effectiveness. It is worth mentioning that it was not possible to estimate the probability of harm and cost avoided in cases of no intervention provided, this might limit the generalizability of the study. Future research proposed could expand the availability of clinical pharmacy medication reviews to other settings such as community pharmacies and primary care clinics and redesign how the reviews are conducted, such as telemedicine and virtual clinics. Also, there is a need to determine whether patients and physician accept the pharmacists’ intervention and services and what targeting mechanism to be employed. Given that the legislative framework to facilitate medication review implementation in community pharmacy is under development, the results of the current study suggest to clinical practice that medication review provided to patients at risk of DRPs is conceivable and that such a service can be quickly implemented. The current study suggests to the scientific community that a variety of cost-avoiding measures and DRP-addressing interventions can aid in promoting the provision of services to address DRPs.

## Conclusion

Clinical pharmacists were heavily involved in resolving DPRs. The most prevalent DRP intervention, was the need for additional diagnostic testing to address the miscellaneous-related DRP. The highest expenditures avoided and spent if DRPs were not detected and addressed were related to the DRP category in appropriate knowledge. The anticipated avoided cost for study outpatient clinic attendance was JOD 1,418,720 per month. DRPs’ high frequency and cost make a compelling justification for additional pharmacist services input by pharmacists in order to improve patient care. More efforts can be put forward to implement services to address DRPs from pharmacists, taking in consideration the full range of settings and innovative techniques, as well as making sure that a viable business model is formed that is accepted by other healthcare professionals and patients themselves.
